# Arachidonic Acid Attenuates Cell Proliferation, Migration and Viability by a Mechanism Independent on Calcium Entry

**DOI:** 10.3390/ijms21093315

**Published:** 2020-05-07

**Authors:** Carlos Cantonero, Jose Sánchez-Collado, Jose J. Lopez, Ginés M. Salido, Juan A. Rosado, Pedro C. Redondo

**Affiliations:** Department of Physiology, Faculty of Veterinary Medicine and Institute of Molecular Pathology Biomarkers (IMPB), University of Extremadura, 10003 Cáceres, Spain; carloscantonero@unex.es (C.C.); josesc@unex.es (J.S.-C.); jjlopez@unex.es (J.J.L.); gsalido@unex.es (G.M.S.); jarosado@unex.es (J.A.R.)

**Keywords:** MDA-MB-231 cells, arachidonic acid, ARC channels, Orai3

## Abstract

Arachidonic acid (AA) is a phospholipase A2 metabolite that has been reported to mediate a plethora of cellular mechanisms involved in healthy and pathological states such as platelet aggregation, lymphocyte activation, and tissue inflammation. AA has been described to activate Ca^2+^ entry through the arachidonate-regulated Ca^2+^-selective channels (ARC channels). Here, the analysis of the changes in the intracellular Ca^2+^ homeostasis revealed that, despite MDA-MB-231 cells expressing the ARC channel components Orai1, Orai3, and STIM1, AA does not evoke Ca^2+^ entry in these cells. We observed that AA evokes Ca^2+^ entry in MDA-MB-231 cells transiently expressing ARC channels. Nevertheless, MDA-MB-231 cell treatment with AA reduces cell proliferation and migration while inducing cell death through apoptosis. The latter mostly likely occurs via mitochondria membrane depolarization and the activation of caspases-3, -8, and -9. Altogether, our results indicate that AA exerts anti-tumoral effects on MDA-MB-231 cells, without having any effect on non-tumoral breast epithelial cells, by a mechanism that is independent on the activation of Ca^2+^ influx via ARC channels.

## 1. Introduction

Changes in the cytosolic Ca^2+^ concentration ([Ca^2+^]_c_) due to Ca^2+^ entry across the plasma membrane have been reported to be involved in cell proliferation [1,2,3,4]. In fact, several cancer cell lines present altered [Ca^2+^]_c_ due to dysregulation of Ca^2+^ entry mechanisms, among others [3,5,6,7,8,9]. The Ca^2+^ currents *I*_SOC_, *I*_CRAC_, and *I*_ARC_ were described as relevant inward Ca^2+^ currents in non-excitable cells including several cancer cell types [10,11]. *I*_ARC_ involves the participation of Orai1α, Orai3, and STIM1, and in several cell types such as HEK293 cells and lymphocytes, the arachidonate-regulated Ca^2+^-selective (ARC) channel conductivity was described to be enhanced by adding low concentrations of arachidonic acid (AA) to the extracellular medium [10,12,13,14,15,16,17]. In addition, the Orai3 N-terminal domain determines the ARC channels selectivity for AA, but not for other fatty-acids [18]. Similarly, the non-metabolizable AA analogue, ETYA, was also shown to be able to activate *I*_ARC_ [19]. Recently, the ARC channel components have been shown to be overexpressed in certain cancer cell types [20,21]. In line with this observation, prostate cancer cells exhibited exacerbated *I*_ARC_ [22]. Cytosolic phospholipase A_2_ (PLA_2_) degrades membrane phospholipids to generate AA and lysophosphatidic acid [23]. Interestingly, PLA_2_ was also reported to be overexpressed in certain cancer cell lines, particularly in the murine breast cancer cell line 4T1 [24] and in basal breast cancer cell lines of human origin such as in MDA-MB-231 and SKBR3 [25,26,27].

The effect of AA on basal breast cancer cells is controversial. While, exogenous AA administration was demonstrated to enhance their proliferation and migration capabilities [27,28], other independent groups have not corroborated these results and, conversely, they have demonstrated that AA metabolites might be involved in cell death evoked by AA administration [29]. The opposite effects observed in the AA-evoked response in the MDA-MB-231 cells were linked to the concentration used [29,30]. Furthermore, AA metabolizing enzymes have also been evidenced to be differently expressed in certain types of cancer cells compared to their respective epithelial control cells [31]. Finally, incubation of MDA-MB-231 cells with high concentrations of AA favored their migration capability [28].

Here, we show that administration of AA (8 µM) to MDA-MB-231 cells reduced cell proliferation and migration. Furthermore, treatment of MDA-MB-231 with AA evoked mitochondrial depolarization and the activation of caspases, thus leading to the activation of apoptosis. Interestingly, AA was unable to evoke changes in [Ca^2+^]_c_, which suggests that MDA-MB-231 cells do not express functional ARC channels.

## 2. Results

### 2.1. Arachidonic Acid (AA) Is Unable to Induce Ca^2+^ Mobilization in MCF10A and MDA-MB-231 Cells

**Figure 1:**
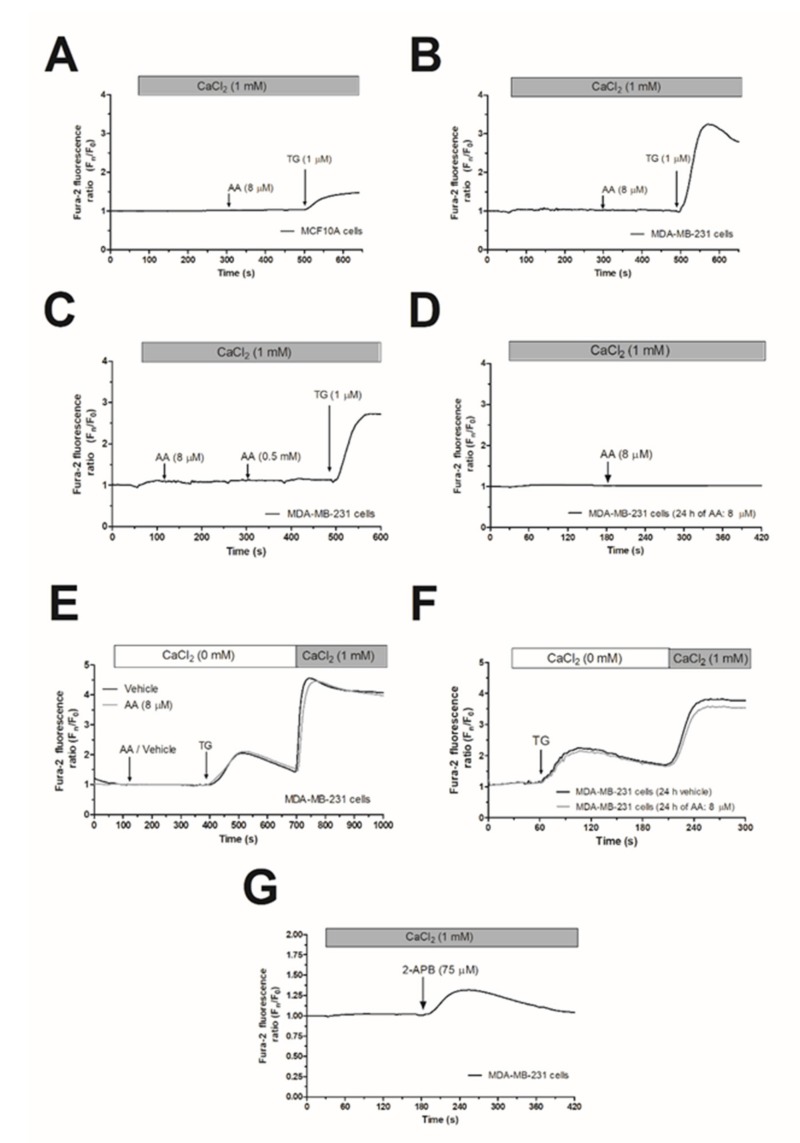
Arachidonic acid (AA) does not evoke changes in [Ca^2+^]_c_ in MCF10A and MDA-MB-231 cells. MCF10A (**A**) and MDA-MB-231 cells (**B–G**) were shed onto coverslips and loaded with Fura-2. Cells were maintained in a medium containing 50 µM of CaCl_2_ and were alternatively excited at 340 and 380 nm and the emission was recorded at 505 nm. (**A–C**) Cells were treated with AA (8 or 500 µM) or thapsigargin (TG, 1 µM) in the presence of extracellular Ca^2+^ (1 mM). (**D**) Cells were cultured with AA (8 µM) for 24 h, and subsequently, they were stimulated with AA (8 µM) in the presence of extracellular CaCl_2_ (1 mM). (**E**) MDA-MB-231 cells were suspended in a Ca^2+^-free HBS medium (100 µM of EGTA was added), then treated with AA (8 µM) or the vehicle, followed by treatment with TG (1 µM); following, CaCl_2_ (1 mM) was added to the extracellular medium 5 min later to visualize Ca^2+^ entry. (**F**) Cells were cultured for 24 h with AA (8 µM), and subsequently, we reproduced similar experimental conditions than the previous one. (**G**) Cells were treated with 2-APB (75 µM) in the presence of extracellular Ca^2+^ (1 mM). Traces are representative of six independent experiments.

In the presence of 1 mM extracellular Ca^2+^, treatment of MCF10A or MDA-MB-231 cells with 8 µM AA was unable to induce significant changes in [Ca^2+^]_c_ (Figure 1A,B; *n* = 6). Subsequent addition of thapsigargin (TG; 1 µM) resulted in an increase in [Ca^2+^]_c_, indicative of Ca^2+^ release and subsequent activation of store-operated Ca^2+^ entry (SOCE; Figure 1A,B). AA was unable to induce changes in [Ca^2+^]_c_ in MDA-MB-231 cells at concentrations as high as 0.5 mM (Figure 1C). In the literature, controversy effects between short and long exposition time-periods to AA have been reported. Therefore, we incubated the MDA-MB-231 cells for 24 h with 8 µM of AA, and subsequently, upon loading cell with Fura-2, they were stimulated with AA (8µM) in the presence of extracellular CaCl_2_ (1 mM), which did not evoke changes in the [Ca^2+^]_c_ (Figure 1D). We have further explored whether treatment with AA might alter SOCE, a major Ca^2+^ entry mechanism in non-excitable cells, whose regulation results are crucial for MDA-MB-231 cell proliferation [5,6]. As depicted in Figure 1E,F, preincubation of MDA-MB-231 cells for 5 min or 24 h with 8 µM of AA had no effect neither in TG-evoked release nor in SOCE in these cells.

In contrast to Orai1 and Orai2, Orai3 might be activated by 2-aminoethoxydiphenyl borate (2-APB), while SOCE is abolished under this experimental condition [32]. In order to test whether MDA-MB-231 cells express functional Orai3, we performed a series of experiments using 2-APB. As depicted in Figure 1G, the addition of 75 µM of 2-APB to MDA-MB-231 cells evoked a transient increase in the [Ca^2+^]_c_ in the presence of extracellular CaCl_2_ (1 mM). This finding suggests the expression of functional Orai3 in MDA-MB-231 cells.

### 2.2. MDA-MB-231 Cells Lack Functional Native Arachidonate-Regulated Ca^2+^-Selective (ARC) Channels

**Figure 2:**
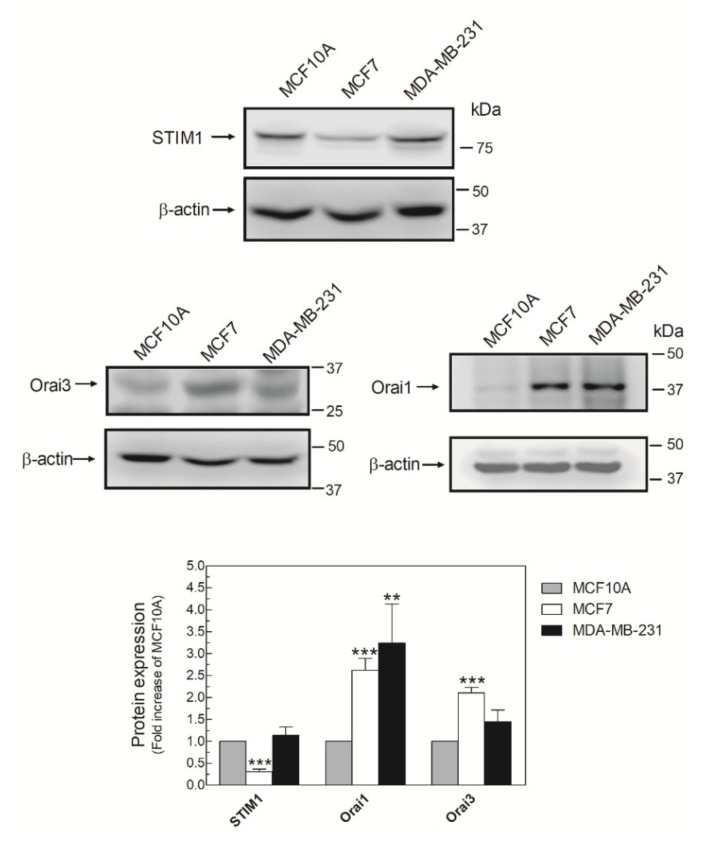
MDA-MB-231 cells express the three components of the arachidonate-regulated Ca^2+^-selective (ARC) channels. MCF10A, MCF7, and MDA-MB-231 cells were seeded in 6-well plates and, upon reaching the adequate cell confluence (90%), they were detached, lysed with NP-40, and denaturated by mixing with Laemmli´s buffer (LB). Subsequent Western blotting (WB) was performed using the anti-STIM1, anti-Orai1, and anti-Orai3 antibodies as described in the Materials and Methods Section. Membranes were reprobed with an anti-β-actin antibody that was used as the loading protein control. Images are representative of 4–6 independent experiments and the histogram represents the fold increase of the protein concentration found with respect to the MCF10A cells. ** *p* < 0.01, *** *p* < 0.001 using ANOVA and Tukey’s post-test, respectively.

It has been described that AA promotes Ca^2+^ entry by interacting with the N-terminal domain of Orai3, which, together with STIM1 and Orai1, forms the ARC channels [15]. Then, we analyzed the expression of the ARC components in MDA-MB-231 cells. As shown in Figure 2, MDA-MB-231 cells expressed the three components of the ARC channels, although the expression of the triad of proteins varied according to the breast cell lines analyzed. Expression of Orai1 was elevated in MDA-MB-231 cells, while the luminal breast cancer cell type MCF7 exhibited high expression of Orai1 and Orai3 and low expression of STIM1 compared with MCF10A (Figure 2).

**Figure 3:**
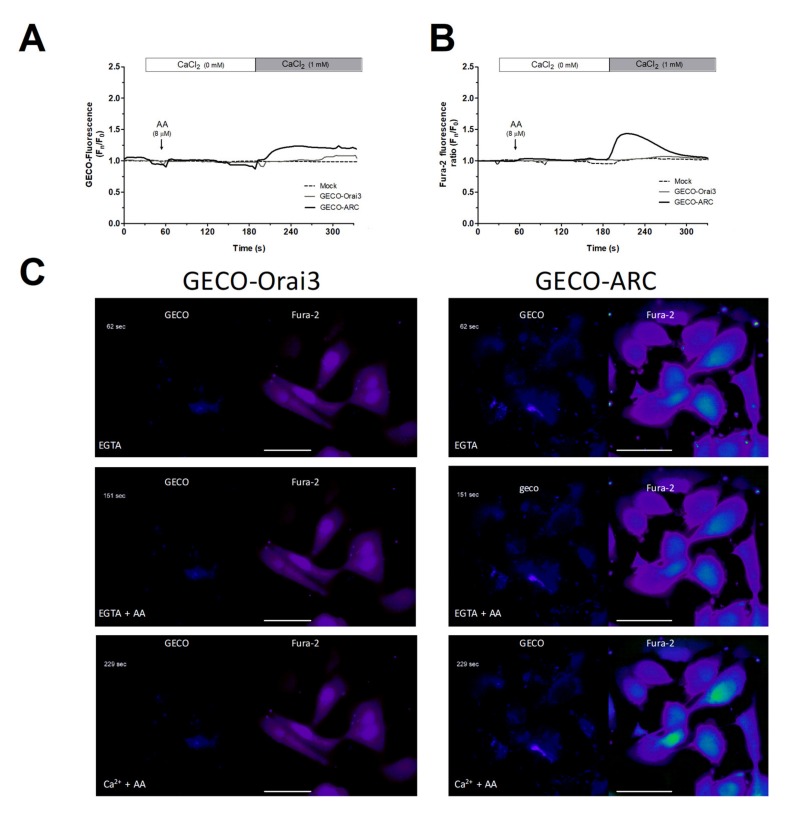
MDA-MB-231 cells artificially expressing ARC channels present AA-evoked changes in the [Ca^2+^]_c_. MDA-MB-231 cells were transfected with either the empty vectors (Mock), the overexpression plasmid of GECO-Orai3, or with the triad of overexpression plasmid for reconstituting the ARC channels (Cherry-STIM1, Orai1-CFP, and GECO-Orai3; GECO-ARC). Upon confirming positive expression of the plasmids using epifluorescence microscopy, cells were maintained in a medium containing 50 µM of CaCl_2_. Cells were alternatively excited at 488 nm (GECO-Orai3 dye; **A** and **C**) and 340/380 nm (Fura-2; **B** and **C**) and fluorescence emitted by the samples was acquired at 505 nm for both fluorescent dyes. At the beginning of the experiments, extracellular medium was supplemented with EGTA (ethylene glycol-bis(β-aminoethyl ether)-N,N,N′,N′-tetraacetic acid; 100 µM) and samples were subsequently incubated for 3 min with AA (8 µM). Next, we added 1 mM of CaCl_2_ to the extracellular medium and we monitored the Ca^2+^ entry evoked by AA stimulation for the next 2 min. Traces are representative of three independent experiments where 2–8 positive transfected cells in each field were analyzed. Bars represent 30 µm.

We further conducted Ca^2+^ experiments in MDA-MB-231 cells transfected with the empty vector (mock) or with overexpression plasmids for GECO-Orai3 alone or Orai1, STIM1, and GECO-Orai3 (GECO-ARC). The use of GECO-Orai3 will allow us to monitor direct Ca^2+^ entry through the channel [17,33]. As depicted in Figure 3, in MDA-MB-231 cells, Ca^2+^ entry evoked by AA was only observed in cells where we efficiently reconstituted the ARC channels (800 ± 200%; *p* < 0.001, *n* = 3), but not in mock or MDA-MB-231 cells transfected with GECO-Orai3 alone. These findings indicate that MDA-MB-231 cells lack functional ARC channels, despite expressing the components that form these channels.

### 2.3. Effect of AA in MDA-MB-231 Cell Proliferation

**Figure 4:**
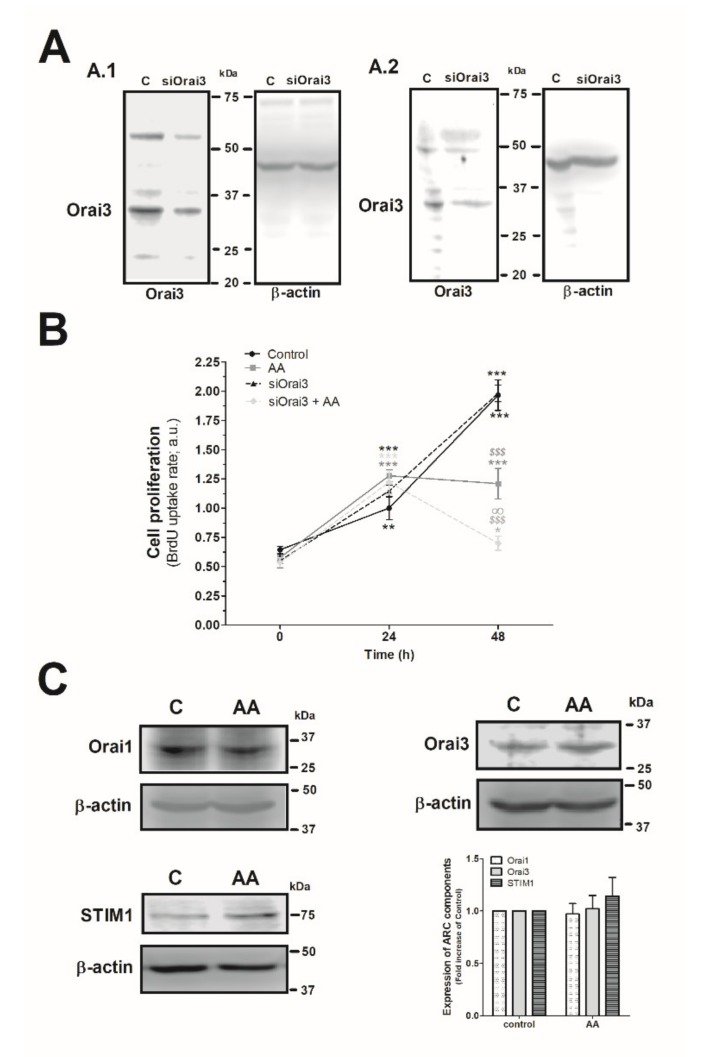
Combination of AA and siOrai3 reduces MDA-MB-231 cell proliferation. (**A**) MDA-MB-231 cells were transfected with the siRNA A (control) or siOrai3 for 48 h (**A.1**) or 96 h (**A.2**), respectively. WB using the anti-Orai3 antibody was done as described in the Materials and Methods, and following, reprobing of the membranes with an β-actin-antibody was conducted for protein loading control. (**B**) MDA-MB-231 cells were transfected with the siRNA A (control and AA) or siOrai3 for 48 h. Then, an equal number of cells were shed in a 96-well plate and were allowed to proliferate in the absence or presence of AA (8 μM). At the indicated time points (0, 24, and 48 h), cells were incubated with BrdU for 2 h. The histogram represents the mean ± S.E.M. (standard error of the mean) of BrdU uptake of eight independent experiments. * *p* < 0.05, ** *p* < 0.01, *** *p* < 0.001 respect the BrdU values found in the control at time 0. *^$$$^ p* < 0.001 with respect to the BrdU values found in the control at each given time point. ^∞^ *p* < 0.05 with respect to the BrdU values found in cells non-genetically modified but treated with AA. We used here the ANOVA and Dunnett’s post-test. (**C**) MDA-MB-231 cells were cultured for 48 h in the presence or absence of AA (8 μM), and cells were subsequently lysed. Upon protein normalization, WB using the anti-STIM1, anti-Orai1, and anti-Orai3 antibodies were performed as described in the Materials and Methods. Bar graph represents the fold increase ± S.E.M. of the protein expression with respect to the control cells non-treated with AA, which did not report statistical significance upon analyzing with ANOVA and Tukey’s post-test.

Next, we explored the effect of AA and Orai3 in MDA-MB-231 cell proliferation. As depicted in Figure 4A, silencing of Orai3 reduced the expression of this protein by 35 ± 15% and 60 ± 6% after 48 h and 96 h of cell transfection, respectively (Figure 4A.1 and 4A.2; *p* < 0.001, *n* = 6). Upon confirming the efficiency of the siOrai3, cells were transfected either with siOrai3 or siRNA A (control) and after 48 h of cell transfection, they were allowed to proliferate for an additional 48 h in the absence or presence of AA (8 µM). Treatment of MDA-MB-231 cells with AA (8 µM) had no effect on cell proliferation during the initial 24 h, but after 48 h significantly attenuated cell proliferation compared to untreated cells (*p* < 0.001, *n* = 8). Interestingly, silencing of Orai3 per se did not significantly modify the proliferation pattern observed in control cells, but significantly attenuated the number of proliferating cells upon stimulation with AA (*p* < 0.001, *n* = 8). These findings suggest that AA plays a negative role in MDA-MB-231 cell proliferation and Orai3 might play a protective effect in these cells. Next, we speculated that AA might be altering the expression of the Orai3 channels, or any the other proteins, but the expression of either Orai1, STIM1, or Orai3 remained unaltered after 48 h of AA administration, as shown in Figure 4C (*p* > 0.05; *n* = 8).

### 2.4. AA Inhibits MDA-MB-231 Cell Migration

**Figure 5:**
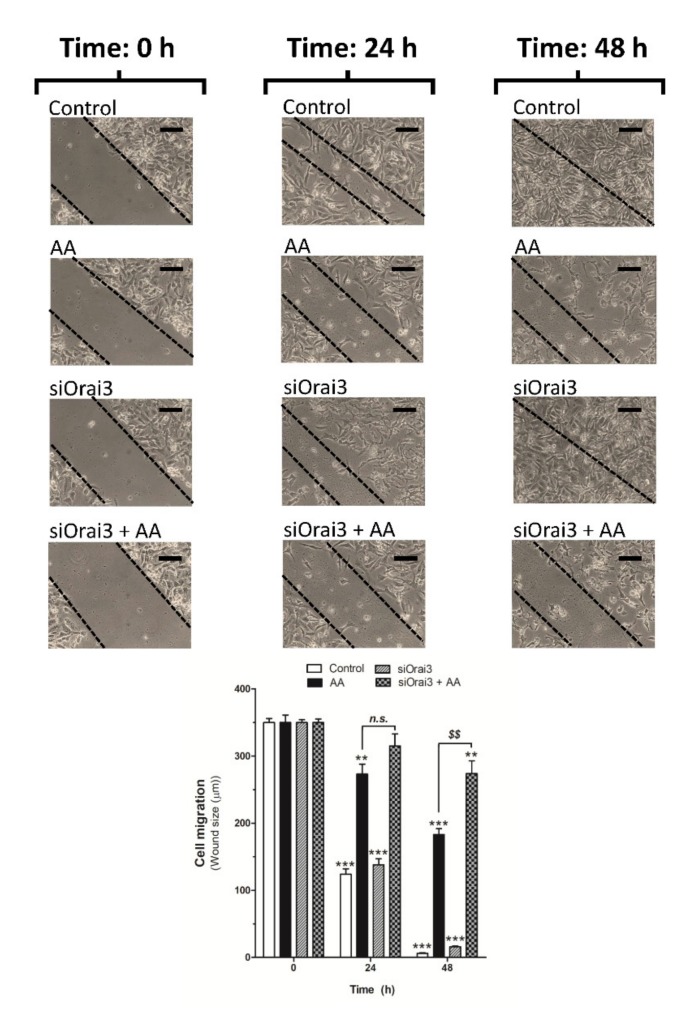
Low AA concentration reduces MDA-MB-231 cell migration. MDA-MB-231 cells were transfected with siRNA A (control and AA) or siOrai3 for 48 h. A scratch was done along the culture dish at time 0 h and cells were allowed to migrate for 48 h in the presence or absence of AA (8 μM). Images of the cell cultures were captured at 0, 24, and 48 h using a bright-field microscope and a 10× objective. Bars represent 100 μm. Bar graph represents the mean ± S.E.M of the wound size expressed in μm. Images are representative of 8–12 images of each condition obtained of three independent experiments. ** *p* < 0.01, *** *p* < 0.001 with respect to the wound size found in control cells at time 0 h. *^$$^ p* < 0.01 with respect to the non-genetically modified but treated with AA. Statistical analysis was done using ANOVA and Dunnett’s post-test.

We further explored the role of AA on cell migration. MDA-MB-231 cells were transfected with siRNA A or siOrai3 and cell migration was monitored as previously described [5,6] at 24 and 48 h. As shown in Figure 5, silencing of Orai3 per se did not affect the ability of MDA-MB-231 cells to migrate, revealing the negligible role of Orai3 in cell migration. Conversely, treatment with 8 μM of AA drastically reduced the migration of both mock or siOrai3 transfected cells at all times evaluated (24–48 h), thus suggesting that AA attenuates MDA-MB-231 cell migration. As reported above, the effect of AA was enhanced in cells transfected with siOrai3, which indicates that Orai3 might attenuate the effect of AA.

### 2.5. AA Attenuates MDA-MB-231 Cell Viability

**Figure 6:**
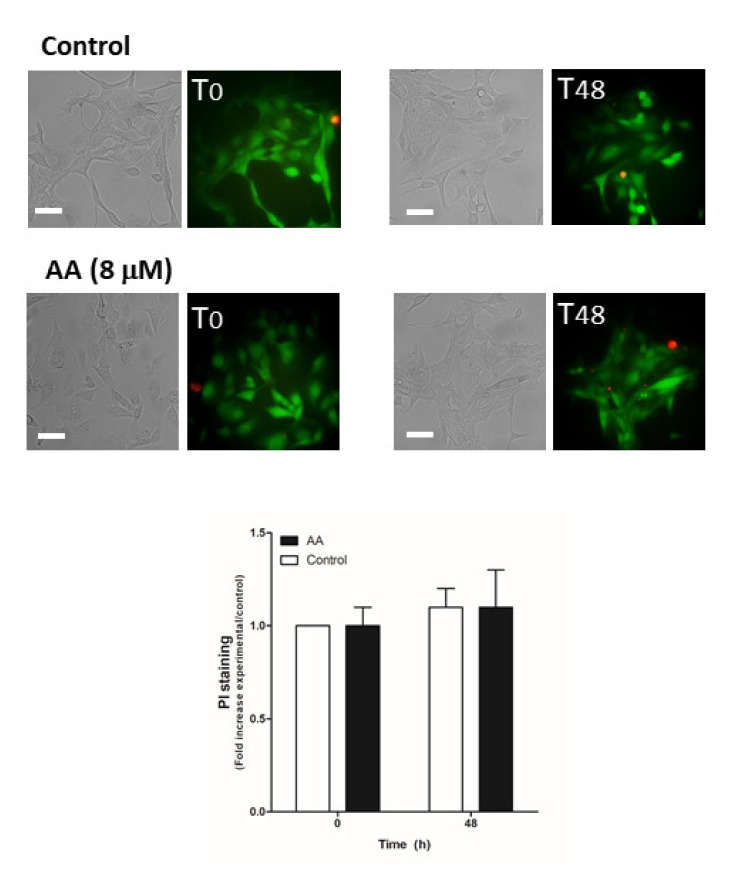
AA does not alter MCF10A viability. MCF10A cells were seeded in 6-well plates and were maintained under regular culture conditions for 48 h in the presence of the vehicle (control) or 8 μM of AA. At the indicated time points, cells were incubated for 45 min with calcein-AM and propidium iodide (PI) (30 min). Images of the cells were taken at 0 and 48 h using an inverted epifluorescence microscope and a 40× oil-objective. Bars represent 30 μm. Bar graph represents the mean ± S.E.M. of the fold increase of PI fluorescence with respect to the control cells at time 0 h, and images are representative of eight different fields from three independent experiments. We used ANOVA and Tukey’s post-test, which reported a *p* > 0.05.

**Figure 7:**
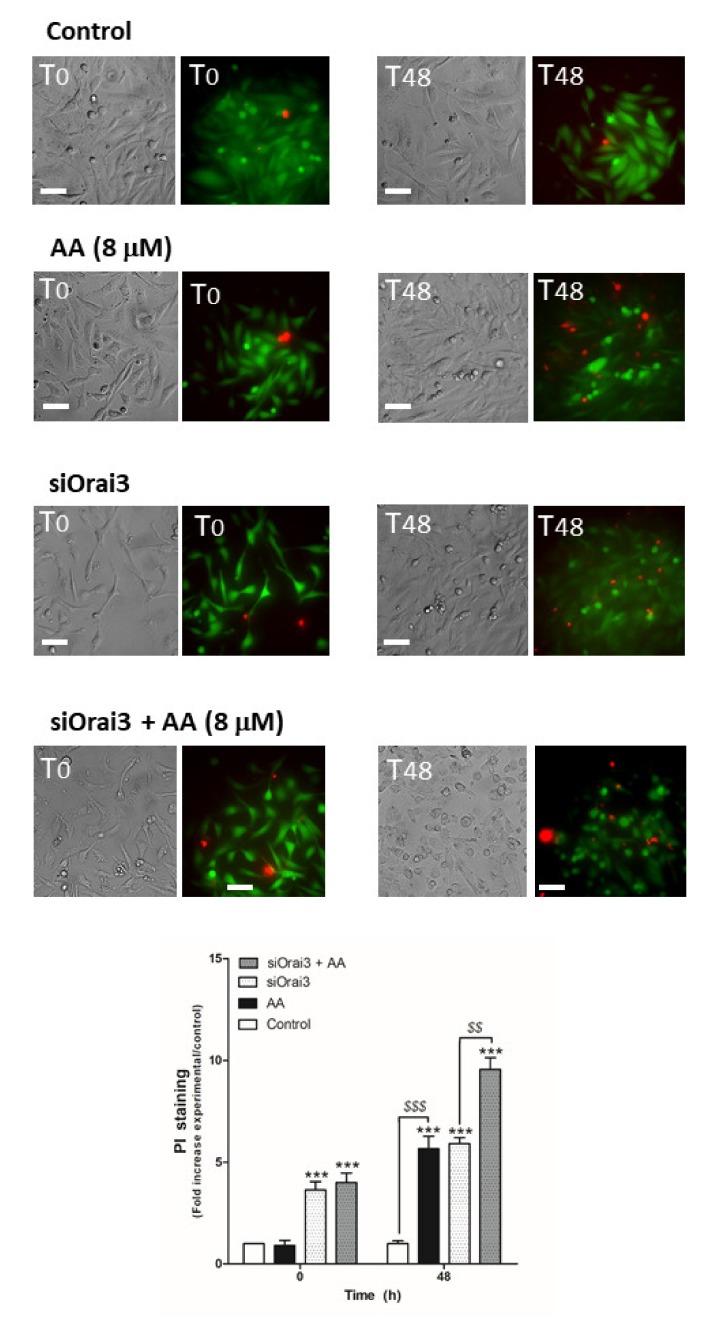
AA evokes cell death in MDA-MB-231 cells. MDA-MB-231 cells were transfected with siRNA A or siOrai3 for 48 h. They were allowed to grow for 48 h in a 6-well plate in the presence of the vehicle (control) or 8 μM of AA. At the indicated time points, extracellular medium was supplemented with calcein-AM (45 min) and PI (30 min). Images were taken at 0 and 48 h using an inverted epifluorescence microscope and a 40× oil-objective. Bars represent 30 μm. Bar graph represents the mean ± S.E.M. of the fold increase of PI fluorescence with respect to the control cells at time 0 h, and images are representative of eight different fields from three independent experiments. *** *p* < 0.001 with respect to the PI fluorescence values found in the control cells at each given time point. ^$$^ *p* < 0.01, ^$$$^ *p* < 0.001 with respect to the PI values found in cells non-treated with AA. We used ANOVA and Tukey’s post-test, which reported a *p* > 0.05.

Next, we evaluated the possible role of AA in cell viability, which may also be responsible for the decrease in cell proliferation and migration observed in MDA-MB-231 cells. MCF10A cells (Figure 6) and MDA- MB-231 cells transfected with siRNA A or siOrai3 (Figure 7) were grown in the absence or presence of AA (8 μM), and at the indicated time points, cell viability was determined as described in the Methods.

As shown in Figure 6, AA did not alter cell viability in MCF10A cells (*p* > 0.05, *n* = 8). Conversely, AA significantly increased the number of positive propidium iodide (PI) MDA-MB-231 cells after 48 h of treatment (Figure 7; *p* < 0.01, *n* = 8). Furthermore, Orai3 silencing per se attenuated cell viability of the MDA-MB-231 cells since the beginning of the experiment. In the presence of AA for 48 h, Orai3 silencing enhanced the effect of AA on MDA-MB-231 cell viability (Figure 7; *p* < 0.01, *n* = 8), thus suggesting that Orai3 exerts certain protective effects in MDA-MB-231 cells.

### 2.6. AA Induces Mitochondrial Membrane Depolarization and Caspase Activation

**Figure 8:**
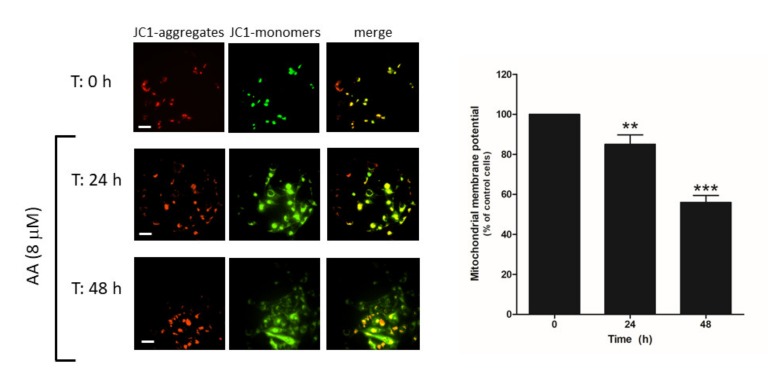
AA disrupts mitochondrial membrane potential in MDA-MB-231 cells. MDA-MB-231 cells were grown in 6-well plate for 48 h in the presence of AA (8 μM). At the indicated times (0, 24, and 48 h), cells were incubated with 2 μM of JC-1 for 30 min. After incubation, images of the cells were obtained by exciting at a 488 nm wavelength and the fluorescence emitted was alternatively recorded at 530 and 580 nm wavelengths. Bars represent 30 μm. Percentages ± S.E.M. of the JC-1 fluorescence ratio (JC-1 aggregates: red, 580 nm)/(JC-1 monomers: green, 530 nm) at the different time points evaluated are represented in the bar graph. Images are representative of four fields from three independent experiments. ** *p* < 0.01, *** *p* < 0.001 with respect to the percentage of JC-1 ratio found in cells at time 0 h. We used ANOVA and Tukey’s post-test, which reported a *p* > 0.05.

Cell death may occur through different mechanisms including apoptosis. Hence, we explored whether AA administration to MDA-MB-231 cells might evoke mitochondrial-dependent activation of apoptosis, as previously reported [29]. MDA-MB-231 cells were grown in the presence of AA for 48 h and, at the indicated time points (0, 24, 48 h), cells were loaded with JC-1, and mitochondrial membrane potential was monitored as described in the Methods. As depicted in Figure 8, the analysis of the changes in the ratio between JC-1 aggregates/monomers indicated that cell treatment for 24–48 h with AA evoked mitochondrial potential depolarization (*p* < 0.001, *n* = 12).

**Figure 9:**
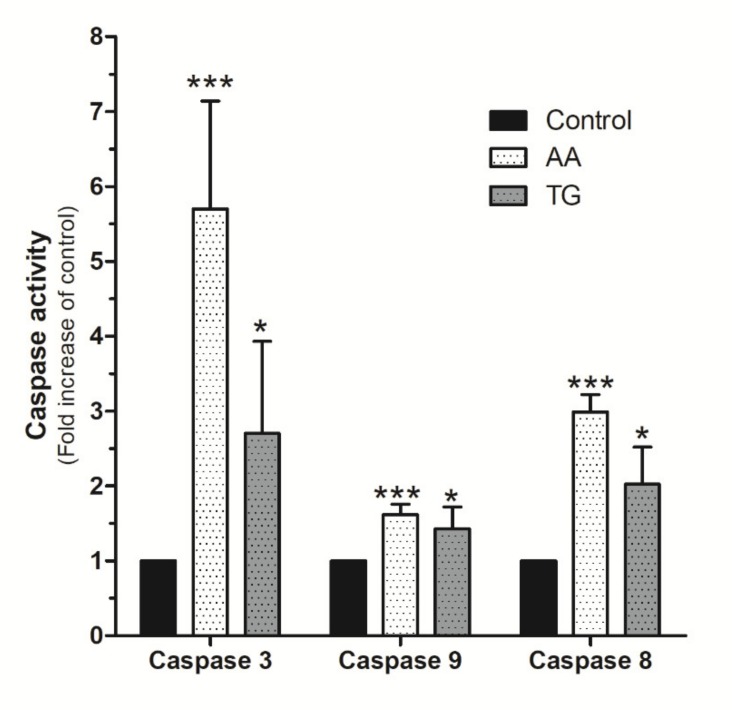
AA activates caspases-3, -8, and -9 in MDA-MB-231 cells. MDA-MB-231 cells were cultured for 48 h, as indicated, in the presence of either the vehicle, 8 μM of AA, or in the presence of 1 μM TG for 24 h. Cells were lysed at 0 h and 24 h or 48 h with ice-cold NP40 buffer. Cell samples were incubated with the different fluorigenic caspase substrates. Fluorescence derived from each caspase activation was recorded using a spectrofluorophotometer and using the wavelengths of 400/505 nm (Ex/Em) or 360/400 nm (Ex/Em), depending whether the caspase substrates were combined with aminomethyl-*coumarin* (AMC) or amino-trifluoromethyl *coumarin* (AFC), respectively. Graph bar represents the changes in the percentage ± S.E.M. of fluorescence emitted by each caspase substrate (a.u.) with respect to control cells considering eight independent experiments. * *p* < 0.05, *** *p* < 0.001 with respect to the values found in control cells. We used ANOVA and Tukey’s post-test, which reported a *p* > 0.05.

Upon confirmation of AA evoked mitochondrial potential disruption in MDA-MB-231 cells, we evaluated the possible activation of caspases by using the specific fluorigenic substrates, as previously reported [6,34,35]. As shown in Figure 9, MDA-MB-231 cells stimulated for 48 h with AA (8 μM) exhibited a substantial and significant increase in caspase-3 activity compared to untreated cells (*p* < 0.001, *n* = 8). Caspase-3 activation by AA was found to be greater than that observed after treatment with TG for 24 h (Figure 9; *p* < 0.05, *n* = 4). Interestingly, AA also induced significant activation of caspases-8 and -9 (Figure 9; *p* < 0.001, *n* = 8).

## 3. Discussion

Triple negative breast cancer is an aggressive subtype of breast cancer that constitutes a therapeutic challenge as it does not respond to inhibitors of estrogen and progesterone receptors. Our results indicate that AA attenuates the ability of MDA-MB-231 cells to proliferate and migrate, and reduces cell viability, probably by the activation of apoptosis. Interestingly, we found that these effects are independent on changes in [Ca^2+^]_c_. This statement is based on the fact that while MDA-MB-231 cells express all the molecular components of the ARC channels, they do not show functional arachidonate-regulated channels and, as a result, AA per se is unable to evoke changes in [Ca^2+^]_c_. Furthermore, AA does not modify SOCE, a major mechanism for Ca^2+^ influx in MDA-MB-231 cells. The possible effect of AA on non-capacitative pathways for Ca^2+^ entry in these cells has not been directly tested, but the expression of SPCA2 in MD-MB-231 cells has been reported to be low [36], and we have not detected any change in the resting [Ca^2+^]_c_. To explain the lack of ARC activation channels, we suppose that the stoichiometry of the ARC channel components (Orai1 and Ora3) is not adequate to generate a functional ARC channel. In fact, it has been described that Orai1 would mainly conduct SOCE in MDA-MB-231 cells, so the Orai1 subunits may be predisposed to constitute SOCE channels in these cells [21]. Furthermore, this idea is also reinforced by the fact that Orai1 and Orai3 are translocated to the plasma membrane upon activation of TRPC6, as our research group has recently demonstrated [5]. Therefore, the components of the ARC channel may be expressed in these cells, but not in the adequate cell location, the plasma membrane, or at right stoichiometry to conform a functional ARC channel [37]. On the other hand, it has also been reported that Orai3 may constitute monomeric capacitative channels in non-small lung adenocarcinoma cells [38], but we discarded this possibility in our cell model according to previously published data by other in MDA-MB-231 cells [21].

It is worth mentioning that, despite the fact that Orai3 channels do not constitute functional ARC channels in MDA-MB-231 cells, we found that 2-APB was able to evoke a sustained increase in [Ca^2+^]_c_, which is indicative of the presence of functional Orai3 channels. According to the literature, 2-APB facilitates Orai3 activation that may contribute to regulate the cytosolic and the endoplasmic reticulum Ca^2+^ contents since Orai3, but not other Orai family members like Orai1 and Orai2, could be associated with IP_3_R in the endoplasmic reticulum membrane in HeLa cells [39]. In fact, the deleterious effects of AA on MDA-MB-231 cells seem to be enhanced upon attenuation of Orai3 expression, thus suggesting that Orai3 might protect MDA-MB-231 cells from the damaging actions of AA. Our observations agree with those found in non-small cell lung adenocarcinoma, where Orai3 was evidenced to regulate cell proliferation and cell cycle progression by activating Akt [38]. Authors have shown that the silencing of Orai3 inhibits Akt, which negatively affects their downstream effector in the cell cycle, the cyclins D1 and D3. Additionally, our results also corroborate those obtained in HeLa and HEK293 cells, where the silencing of Orai3 downregulated cell proliferation by a Ca^2+^ independent mechanism [40]. A more recent publication claimed that Orai3 evokes downregulation of p53 in ER+ breast cancer cells, which involves the activation of the PI3K/SgK-1/SeK-1 pathway [41].

Our findings concerning the role of AA in MDA-MB-231 cell proliferation are in agreement with previous studies [29], although they are inconsistent with others [30]. This discrepancy might be attributed to different experimental approaches such as the different AA concentrations or stimulation times used in the diverse studies, which might lead to the activation of distinct signaling molecules. In this sense, we did not find differences in [Ca^2+^]_c_ with short or long incubation time-periods (see Figure 1). However, other studies, for instance, have analyzed different AA concentrations, finding that the peroxisome proliferator-activated receptor-alpha (PPARα) was reported to be more efficiently activated by 10 μM of AA [30], and modulates cell proliferation and senescence in breast cancer cells through the regulation of carnitine palmitoyltransferase 1C (CPT1C) [42].

Most of the effects of AA on MDA-MB-231 cell proliferation and migration might be attributed to the activation of apoptotic events in these cells. AA has been shown to induce apoptosis in a variety of cancer and non-tumoral cells including human melanoma cell lines, vascular smooth muscle cells, tumor brain cells, and cultured spinal cord neurons [43,44,45,46]. In culture, spinal cord neuron treatment with 10 µM AA is able to induce activation of caspases-3, -8, and -9 [46]. We observed similar results in MDA-MB-231 cells, in addition to the disruption of the mitochondrial membrane potential. AA deleterious effects on mitochondrial function have previously been reported and, in the murine fibroblast cell line (C3HA), AA evokes mitochondrial potential disruption by directly affecting the mitochondrial permeability transition pore, independently of the mitochondrial Ca^2+^ content [47,48]. The latter, together with the activation for caspase-8, might trigger the activation of caspase-9, which, in turn, leads to the activation of downstream caspases such as caspase-3.

Altogether, our data reveal a negative role of AA in the cancer hallmarks of the MDA-MB-231 triple negative breast cancer cells, and these observed harmful effects of AA are exacerbated when Orai3 expression is silenced.

## 4. Materials and Methods

### 4.1. Material and Cell Lines

AA was ordered from Abcam plc., which provided us with batches of AA solved in ethanol (Cambridge, UK; catalogue number: Ab120916). Bromodeoxyuridine (BrdU) cell proliferation kit was ordered from BioVision Inc. (Milpitas, CA, USA). DharmaFECT Kb transfection reagent was obtained from Dharmacon Inc. (Lafayette, CO, USA). G-GECO1-Orai3 was a gift from Michael Cahalan (Addgene plasmid #73563; http://n2t.net/addgene: 73563; RRID:Addgene_73563) [33]. Orai1-CFP was a gift from Anjana Rao (Addgene plasmid #19757; http://n2t.net/addgene: 19757; RRID:Addgene_19757) [49]. pENTR1a-mCherry-STIM1 was a gift from Nicolas Demaurex (Addgene plasmid #114176; http://n2t.net/addgene:114176; RRID:Addgene_114176) [50]. Horseradish peroxidase-conjugated goat anti-mouse immunoglobulin G (IgG) antibody, and goat anti-rabbit IgG antibody were from Jackson Laboratories (West Grove, PA, USA). SuperSignal^®^ West Dura extended duration substrate reagent was from ThermoFisher Scientific (Waltham, MA, USA). JC-1 and Fura-2 acetoxymethyl ester (Fura-2/AM) were from Molecular Probes (Leiden, Netherlands). Mouse monoclonal anti-STIM1 antibody (catalogue number 6140954, epitope: amino acid 25-139) was purchased from BD-Bioscience^®^ (Madrid, Spain). Mission^®^siRNA human Orai3 (siRNA1; catalogue number EHU131741 and previously used by others [39]), siRNA A, rabbit polyclonal anti-Orai1 antibody (catalogue number O8264, epitope: amino acids 288–301 of human Orai1), mouse monoclonal anti-Orai3 antibody (Clone 1B4F1, catalogue number, epitope: 19 amino acids of the C-Terminal domain), rabbit polyclonal anti-β-actin antibody (catalogue number A2066, epitope: amino acids 365–375 of human β-actin), caspase-3, -8, and -9 fluorigenic substrates, thapsigargin (TG), EGTA (ethylene glycol-bis(2-aminoethylether)-N,N,N′,N′-tetraacetic acid), HEPES (4-(2-hydroxyethyl)piperazine-1-ethanesulfonic acid), and bovine serum albumin (BSA) as well as other reagents of analytical grade were from Sigma (Madrid, Spain).

MCF7 and MDA-MB-231 cells were collected from the ATCC^®^ collection (Manassas, VA, USA); meanwhile, MCF10A cells were gently provided by Potier-Cartereau (Université Francois Rabelais, Tours, France). Breast cancer cell lines were cultured using DMEM supplemented with 10% (*v*/*v)* fetal bovine serum (FBS) and 100 U/mL penicillin/streptomycin; while non-tumoral cells, MCF10A, were cultured using DMEM-F12 supplemented with 10% (*v*/*v)* horse serum, 10 µg/mL insulin, 0,5 mg/mL hydrocortisone, 20 ng/mL EGF, 100 ng/mL cholera toxin, and 100 U/mL penicillin/streptomycin. Both cell types were cultured in an incubator at 37 °C with 5% CO_2_.

### 4.2. Determination of the Changes in The Cytosolic Free-Ca^2+^ Concentration ([Ca^2+^]_c_)

MCF10A and MDA-MB-231 cells were shed onto coverslips (1 × 10^5^ cells/mL), as previously described [6], and the next day, they were incubated for 30 min at 37 °C in the presence of 2 μM of Fura-2/AM. Upon removing the excess of the Ca^2+^ dye by replacing the medium with fresh HBS medium (containing 50 μM of CaCl_2_), cells were alternatively excited at 340/380 nm and the emitted fluorescence was acquired at 505 nm by using an inverted fluorescence microscope and 40× objective. Changes in the Fura-2 fluorescence were monitored while the cells were treated with either AA (8 or 500 μM), TG (1 μM), or 2-APB (75 μM). Fluorescence ratio (F340/F380) was calculated pixel by pixel, and the data are presented as F_n_/F_0_, where F_n_ is the experimental Fura-2 340/380 fluorescence ratio and F_0_ is the mean basal Fura-2 340/380 fluorescence ratio.

Additionally, we took advantage of the G-GECO1-Orai3 (from now, GECO-Orai3) properties to analyze the Ca^2+^ entry through ARC channels evoked by AA. Cell transfection for 24 h with the triad of STIM1, Orai1, and Orai3 overexpression plasmids was previously described to efficiently reconstitute ARC channels; meanwhile, GECIs-fused to Ca^2+^ channels constructs, like GECO-Orai3, have been documented to be excellent tools to monitor Ca^2+^ entry through a particular channel, since fluorescence of the GECIs-Ca^2+^ dyes drastically increases only when Ca^2+^ actually passes through the channel [17,33]. Therefore, MDA-MB-231 cells were shed onto coverslips inside a 6-well plate, and on the following day, cells were transfected with either the empty vectors or with the GECO-Orai3, Orai1-CFP, and Cherry-STIM1 overexpression plasmids or with the GECO-Orai3 overexpression plasmid alone. After 24 h, cells were incubated with Fura-2/AM, as described above. The day of the experiments, cells were maintained in a Ca^2+^-free HBS-medium (100 μM of EGTA was added) and alternatively excited at 340/380 nm to monitor Fura-2 fluorescence or at 488 nm to visualize GECO-Orai3 emitted fluorescence. Fluorescence emitted by both dyes was recorded at 505 nm using an inverted fluorescence microscope and 100× oil-objective.

### 4.3. Western Blotting (WB)

MCF10A and MDA-MB-231 cells were lysed with ice-cold NP-40 buffer. Later, proteins were denaturated by mixing with Laemmli’s Buffer (LB, 5% of dithiothreitol) and they were then suggested to SDS-PAGE (sodium dodecyl sulfate-polyacrylamide gel electrophoresis; 10% acrylamide/bis-acrilamide) and, subsequent WB was performed using specific anti-STIM1 (diluted 1:500 in blocking buffer for 2 h), anti-Orai3 (1:1000 in blocking buffer overnight), anti-Orai1 (diluted 1:1000 in blocking buffer for 2 h), and anti-β-actin (diluted 1:1000 in blocking buffer for 1 h), as previously described [5]. Optic densitometry of the resulting membranes was acquired with a C-digit LICOR^®^ and was analyzed with the free-software ImageJ from NIH (Bethesda, MD, USA).

### 4.4. Cell Proliferation Assay

At the beginning of the experiments, 5 × 10^3^ cells/mL were shed in a 96-well plate and were allowed to proliferate for 24 h until reaching the optimal cell confluence (60–80 %). Following this, cells were transfected for 48 h with siRNA A (control) or with siOrai3 [15,39]. Later, cells were treated for an additional 48 h with either the vehicle or AA (8 μM). At the indicated time points (0, 24, 48 h), cells were incubated with BrdU for additional 2 h and were subsequently fixed. Proliferation was determined following the manufacturer’s instructions and using the BrdU kit from VioBiosion Inc. (Milpitas, CA, USA). Absorbance in samples was measured at 450 nm using a plate reader (Epoch, Biotek, Swindon, UK).

### 4.5. Migration Assay

MDA-MB-231 cell migration was analyzed by using the wound healing assay as described elsewhere [5,6]. Briefly, both cells types were transfected with either siRNA A or siOrai3 for 48 h and, after corroborating the efficiency of the transfections, cells were allowed to proliferate until reaching the maximum levels of confluence. At time 0 h, a scratch was done in the culture dishes using a pipette tip with a 90° angle. Following this, both cell types were grown for 48 h in the absence or presence of AA (8 μM); meanwhile, pictures of the cultures were taken at 0, 24, and 48 h using a bright-field microscope and 10× objective. Data were expressed as the mean ± S.E.M. of the wound size in μm to compare between the different treatments.

### 4.6. Cell Death and Mitochondrial Potential Depolarization Analysis

MCF10A and MDA-MB-231 were transfected with siRNA A or siOrai3 as indicated. After 48 h of cell transfection, cells were treated either with the vehicle or with AA (8 μM) for additional 48 h. At the indicated time points (0 and 48 h, respectively), cells were incubated with calcein-AM (2 μM) for 45 min and, the for the last 30 min, the extracellular medium was supplemented with 4 μM of propidium iodide (PI). Once incubation time was over, images of the middle cell plane were acquired using an inverted fluorescence microscope and a 40× WD objective. Cell death evoked as a consequence of AA treatment was determined by considering the increase of the PI fluorescence with respect to the values found in the control cells at the indicated time points.

Additionally, changes in the fluorescence of JC-1 were analyzed in order to determine whether AA might evoke mitochondrial potential depolarization. Thus, MDA-MB-231 cells were grown for 48 h in the presence of AA (8 μM) and, at the indicated times (0, 24, and 48 h), cells were subsequently incubated for an additional 30 min with 2 μM of JC-1 [51]. Finally, JC-1 was excited at a 488 nm wavelength and cell images were acquired at the emission wavelengths of 530 nm (JC-1 monomers, “JC-1 green”) or 580 nm (JC-1 aggregates, “JC-1 red”) using an inverted fluorescence microscopy and 40× WD objective. Ratio of JC-1 green/JC-1 red was used to compare mitochondrial membrane polarization along the experiment.

### 4.7. Analysis of Caspase Activity

MDA-MB-231 cells were shed at equal concentration (5 × 10^6^ cells/mL) and were cultured for 48 h in the absence or presence of AA (8 μM). Cells were subsequently lysed using ice-cold NP-40 buffer for 30 min and stored at −20 °C. Then, 200 μL of cell lysates were mixed with 400 μL of reaction buffer (supplemented with 20 μM of the appropriated fluorigenic caspase substrates) and were incubated at 37 °C for 2 h. Caspase activities were finally determined using a spectrofluorophotometer by exciting the samples at either 400 nm (substrate of caspase-3; Z-DEVD-AFC) or 360 nm (substrate of caspase-8 and -9; AC-VETD-AMC, and AC-LEHD-AMC, respectively). The fluorescence emitted was recorded at 505 nm (substrate of caspase-3) or 400 nm (substrates of caspase-8 and -9). Data were represented as the fold increase ± S.E.M. of the respective caspase substrate-derived fluorescence with respect to the fluorescence values found in resting cells.

### 4.8. Statistical Analysis

Analysis of data using Student’s *t*-tests was done for establishing the statistical significance between the control and treatment groups. ANOVA analysis of the variance and subsequent Tukey’s or Dunnett’s post-tests were done for multiple comparisons. *p* < 0.05 was considered as statistically significant.

## 5. Conclusions

In summary, the treatment of MDA-MB-231 cells with 8 µM AA leads to the attenuation of cell proliferation and migration that may be attributed to the activation of apoptosis and decrease in cell viability. Our observations indicate that the effect of AA on MDA-MB-231 cells is independent of changes in [Ca^2+^]_c_ evoked by AA directly or to the modification of SOCE in these cells. Interestingly, while MDA-MB-231 cells do not exhibit functional ARC channels, Orai3 seems to play a protective effect against the harmful effects of AA in these cells. As AA exerts its deleterious effects, specifically on tumoral cells and not in non-tumoral breast epithelial cells, AA might be further investigated as a potential anti-tumoral agent for the treatment of triple negative breast cancer.

## Abbreviations

AA: Arachidonic acid; ARC, arachidonate-regulated Ca^2+^-selective channels; BrdU, Bromodeoxyuridine; [Ca^2+^]_c_, cytosolic Ca^2+^ concentration; CPT1C, carnitine palmitoyltransferase 1C; EGTA, ethylene glycol-bis(2-aminoethylether)-N,N,N′,N′-tetraacetic acid; PI; PLA_2_, phospholipase A_2_; PPARα, peroxisome proliferator-activated receptor-alpha; TG, thapsigargin.
